# Blackout and supply chains: Cross-structural ripple effect, performance, resilience and viability impact analysis

**DOI:** 10.1007/s10479-022-04754-9

**Published:** 2022-06-03

**Authors:** Dmitry Ivanov

**Affiliations:** Berlin School of Economics and Law, Department of Business Administration, Supply Chain and Operations Management, 10825 Berlin, Germany

**Keywords:** Supply chain, Disruption, Resilience, Blackout, Power outage, Simulation, Digital twin, Viability, Ripple effect, Structural dynamics

## Abstract

Increased electricity consumption along with the transformations of the energy systems and interruptions in energy supply can lead to a blackout, i.e., the total loss of power in an area (or a set of areas) of a longer duration. This disruption can be fatal for production, logistics, and retail operations. Depending on the scope of the affected areas and the blackout duration, supply chains (SC) can be impacted to different extent. In this study, we perform a simulation analysis using anyLogistix digital SC twin to identify potential impacts of blackouts on SCs for scenarios of different severity. Distinctively, we triangulate the design and evaluation of experiments with consideration of SC performance, resilience, and viability. The results allow for some generalizations. First, we conceptualize blackout as a special case of SC risks which is distinctively characterized by a simultaneous shutdown of several SC processes, disruption propagations (i.e., the ripple effect), and a danger of viability losses for entire ecosystems. Second, we demonstrate how simulation-based methodology can be used to examine and predict the impacts of blackouts, mitigation and recovery strategies. The major observation from the simulation experiments is that the dynamics of the power loss propagation across different regions, the blackout duration, simultaneous unavailability of supply and logistics along with the unpredictable customer behavior might become major factors that determine the blackout impact and influence selection of an appropriate recovery strategy. The outcomes of this research can be used by decision-makers to predict the operative and long-term impacts of blackouts on the SCs and viability and develop mitigation and recovery strategies. The paper is concluded by summarizing the most important insights and outlining future research agenda toward SC viability, reconfigurable SC, multi-structural SC dynamics, intertwined supply networks, and cross-structural ripple effects.

## Introduction

Supply chains (SC) are multi-structural systems composed of organizational, informational, financial, technological, process, product and energy structures (Ivanov 2018). As every complex system, SCs are exposed to uncertainty and risks. Literature has developed a profound body of knowledge about disruption risks in SCs, e.g., earthquakes, fires, strikes, pandemics (Aldrighetti et al. 2021, Altay et al. 2018, Dubey et al. 2021b, Hosseini et al. 2019, Queiroz et al. 2020). Performance impact analysis, mitigation and recovery strategies have been extensively studied, mostly concerning the organizational SC structure, e.g., critical supplier identification and back-up supply recovery (Baghersad et al. 2021, Bode et al. 2011, Chopra et al. 2021, Demirel et al. 2019, Dolgui et al. 2020a, Dubey et al. 2019, Ivanov 2021d, Lücker er al. 2021, Sanci et al. 2021). Some works focused on disruptions in the information structure such as cyber-attacks (Sawik 2020). However, disruptions in the energy structures still represent a research gap.

A blackout is the most severe form of power losses characterized by total loss of power in an area (or a set of areas) of a longer duration. Examples include power outage in Texas in February 2021 with the loss of a large part of electrical power (Bloomberg 2021) and provinces Heilongjiang, Jilin and Liaoning in China in 2021 leading to severe consequences for society viability and SC resilience (Disis 2021). Busby et al. (2021) point to economic losses from lost output and damage are estimated to be $130 billion in Texas alone. Increased electricity consumption along with the transformations of the energy systems make the blackout to one of the most likely and dangerous SC disruption risks for very near future (Emenike and Falcone 2020). An informal survey conducted by us with SC managers in September 2021 showed that they fear the total blackout more as pandemics or other severe crises. Later, geopolitical tensions in Spring 2022 led to the increased risks of energy supply interruptions at the global scale exposing material flows in SCs to disruptions.

Adversely, the energy shortage-triggered material shortages and delivery delays can propagate downstream the SC, causing the *ripple effect* and performance degradation in terms of revenue, service level and productivity decreases (Dolgui et al. 2018, Ghadge et al. 2021, Gholami-Zanjani et al. 2021, Li et al. 2021, Llaguno et al. 2021, Park et al. 2021, Shi et al. 2021). One can expect simultaneous ripple effects, i.e., propagation of the power outage and propagation of disruptions in material flows. Moreover, disruptions in the SC energy structure can influence not only the organizational structure due to disrupted material flows (e.g., unavailability of warehouses) but also propagate to other structures (e.g., financial structure due to missing electronic payments and information structure due to disruptions in the digital SC).

The blackout can impact not only resilience of individual SCs but viability of the whole business ecosystems. As pointed in Ivanov and Dolgui (2020), Ivanov (2020b) and Ruel et al. (2021), viability is the SC ability to survive through the severe crisis and so securing the viability of critical ecosystems (e.g., communication, mobility, and food) responsible for provision of society with goods and services, echoed by Nasir et al. (2021) and Wang and Yao (2021). The blackout is a distinct type of SC disruptions that affects both the SC performance and ecosystem viability.

In this study, we perform a simulation analysis using anyLogistix digital SC twin to identify potential impacts of blackouts on SCs for scenarios of different severity. We examine SC dynamic behaviors under blackout conditions for several scenarios. The outcomes of this research can be used by decision-makers to predict the operative and long-term impacts of blackouts on the SCs and product availability and develop mitigation and recovery strategies.

The contribution of this study is twofold. First, we conceptualize blackout as a special case of SC risks which is distinctively characterized by a simultaneous shutdown of SC processes, disruption propagations (i.e., the ripple effect), and danger of viability losses for entire ecosystems. Second, we demonstrate how simulation-based methodology can be used to examine and predict the impacts of blackouts. Distinctively, we design experiments and analyse the results with consideration of three dimensions, i.e., SC performance, resilience, and viability. A set of sensitivity experiments allows illustrating the model’s behavior for different blackout scenarios along with its value for decision-makers. The major observation from the simulation experiments is that the dynamics of the power loss propagation across different regions, the blackout duration, simultaneous unavailability of supply and logistics along with the irrational customer behavior might become major factors that determine the blackout impacts.

The rest of this paper is organized as follows. In Sect. 2, we present the underlying case-study and simulation model. Section 3 describes the modelling environment. The experimental setup and results are shown in Sect. 4. The Sect. 5 discusses managerial implications as well as the future research directions. The paper is concluded in Sect. 6 by summarizing the most important insights and outlining future research agenda toward SC viability, reconfigurable SC, multi-structural SC dynamics, and cross-structural ripple effects.

## Case-study

**Fig. 1:**
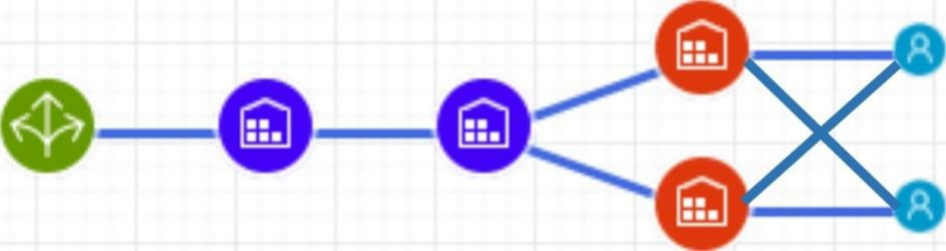
Supply chain design

We examine dynamic behaviour of an SC with a homogenous product of everyday need with quite a stable demand under rational customer behaviour (i.e., if no panic buying occurs). The SC comprises of a factory, an upstream CDC (central distribution center), a downstream CDC, two regionals distribution centres (RDC), and 50 customers (Fig. 1).

The 50 customers order every 7 days a total demand of 8,988 units per order cycle. To avoid randomness in the output analysis and without loss of generality, we allow for deterministic demand which ranges from 70 units to 1667 units depending on the customer. The lead time between factory and upstream CDC is 4 h; between the upstream and downstream CDCs – 42 h; between downstream CDCs and RDCs – 2–10 h; and between RDCs and customers – 1–10 h. The RDCs, CDCs, and factory are located in different regions each of which has its own electricity network; however, the networks are interconnected and a blackout in one of the networks can propagate to the network of another region and cause a blackout there.

**Fig. 2:**
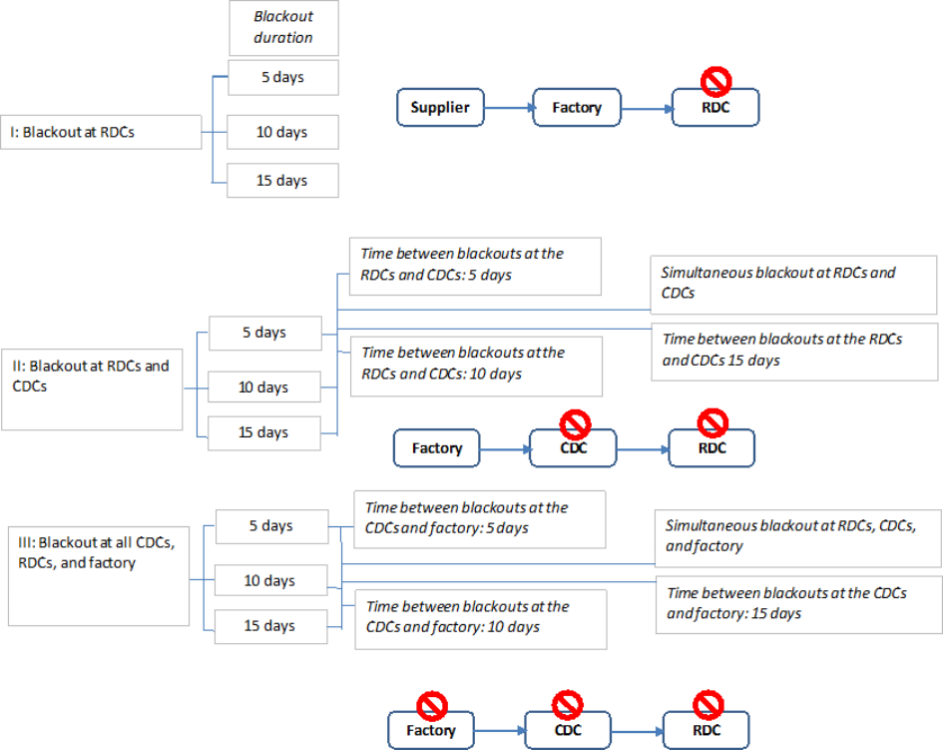
Blackout scenarios

We consider the following blackout scenarios (Fig. 2).

Blackouts can have short (5 days), medium (10 days) and long (15 days) durations. We note that the re-order frequency in our case study is 7 days. This number is a usual business practice. We consider both localized blackouts downstream at the RDCs, simultaneous blackout at all the SC echelons, and blackout propagation from the RDC’s region upstream to CDCs and the factory with different speed and of different duration. In addition, we account for irrational consumer behaviour in a case of a blackout in anticipation of shortages resulting in demand increase during the blackout period of 200%. This is in line with observations done at the beginning of the COVID-19 pandemic and the associated panic buying (Ardolino et al., 2021; Choi, 2021; Paul & Chowdhury, 2021). In total, our setting leads to 54 different scenarios for analysis (see Table 1).

## Model

### Modelling environment and control logic

Our model is created and solved in anyLogistix simulation and optimization toolkit which represents a digital SC twin. In anyLogistix, the SC has been designed by defining all the locations (factory, warehouses), customers, demand, inventory, sourcing and shipment control policies, costs, revenues, and disruption events (Ivanov, 2019; Singh et al., 2021; Burgos & Ivanov, 2021). The simulation methodology has been recognized as an important tool to study SC dynamics under disruptions (Macdonald et al., 2018, Ivanov 2020a, Li et al., 2020, Ivanov 2021b, Zhao et al., 2019).

**Fig. 3:**
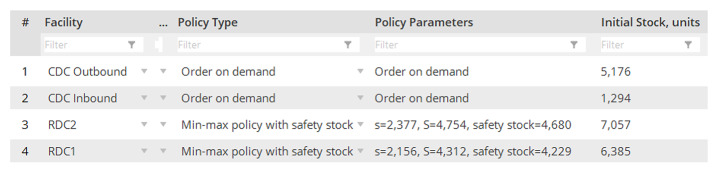
Inventory control policy data

**Fig. 4:**
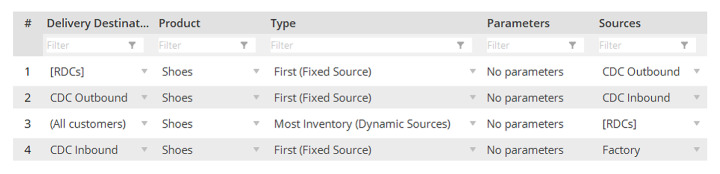
Sourcing control policies

The following control policies have been used for experiments (Figs. 3 and 4).

The inventory control is based on an OUT (order-up-to-level) policy with some re-order point (s) and target inventory (S), and some safety stock (Disney et al., 2020; Boute et al., 2021). The upstream sourcing is a linear system with fixed sources, while the downstream sourcing from RDCs to customers is based on the Most Inventory (Dynamic Sources) rule meaning that the fulfilment of the next incoming order is planned at the RDC with the currently highest inventory level. The backordering is allowed (Schmitt et al., 2017).

### Performance indicators

For analysis, we use the following performance indicators in line with studies by (Dolgui et al., 2020a; Hosseini & Ivanov, 2021; Namdar et al., 2021; Singh et al., 2021):

- Financial SC performance – profit,
- Customer performance – ELT (expected lead time) service level,
- Operational performance – alpha service level.

The profit is computed as a difference between the total revenue and total SC costs which include material, production, transportation, inventory holding and fixed facility costs. The ELT service level and alpha service levels are computed according to Eqs. (1) and (2), respectively.

1 $$ELT SL=\frac{{O}_{on-time}}{{O}_{out}}$$;

2 $$Alpha SL=\frac{{O}_{available}}{{O}_{total}}$$,

where *O*_*on−time*_ is the number of on-time delivered orders at customers, i.e., the number of orders that were delivered within the ELT. In our model, ELT is 2 days for all customers;

*O*_*available*_ is number of successful orders, i.e., the number of orders that were delivered from stock available at the RDCs at the moment of the order placement;

*O*_*out*_ is the number of all outgoing orders including on-time and delayed orders;

*O*_*total*_ is the number of all orders placed at the RDCs.

The alpha service level shows the estimation of the number of unsuccessful orders. The unsuccessful orders are the placed orders requiring the quantity of products that is not available at the warehouse at the time when this order is placed, and the dropped orders. The alpha service level is the product availability indicator. The ELT service level shows the fraction of on-time orders delivered at customers and is the on-time delivery indicator.

Since the blackout has both economical and societal impacts, we consider profit as SC performance indicator, alpha service level as SC resilience indicator, and the ELT service level as viability indicator.

## Experiments

In this section, we present our experimental results and analyse them according to different blackout scenarios and SC reactions.

### Experimental design

We design our experimental environment to examine the SC performance and product availability in case of singular, simultaneous and propagated blackouts of different severities subject to answering the following questions:

- What is the impact of blackouts on the SC financial and operational performance from the resilience point of view?
- What is the impact of blackouts on the product availability from the viability point of view?
- What is the role of the scope and timing of blackout propagations?
- What are the most critical scenarios of blackouts?
- What is the impact of irrational (panic) customer behaviors in the wake of a blackout?

Organization of the experiments is as follows. For analysis, we consider two groups of scenarios, i.e. sequential and simultaneous blackouts. In each of these two groups, we further diversify our analysis including both rational and irrational (i.e., panic buying) customer behaviors during the blackout periods. Finally, we compare the SC reactions in different cases and draw conclusions on the blackout impacts on the SC performance, resilience and viability. For verification, tracking of the simulation runs, analysis of output log files, and visualization analysis were used. For testing, we use replications in comparison and variation experiments. A warm-up period of two months prior to the disruption (i.e., the blackout) is considered.

### Experiments

**Fig. 5:**
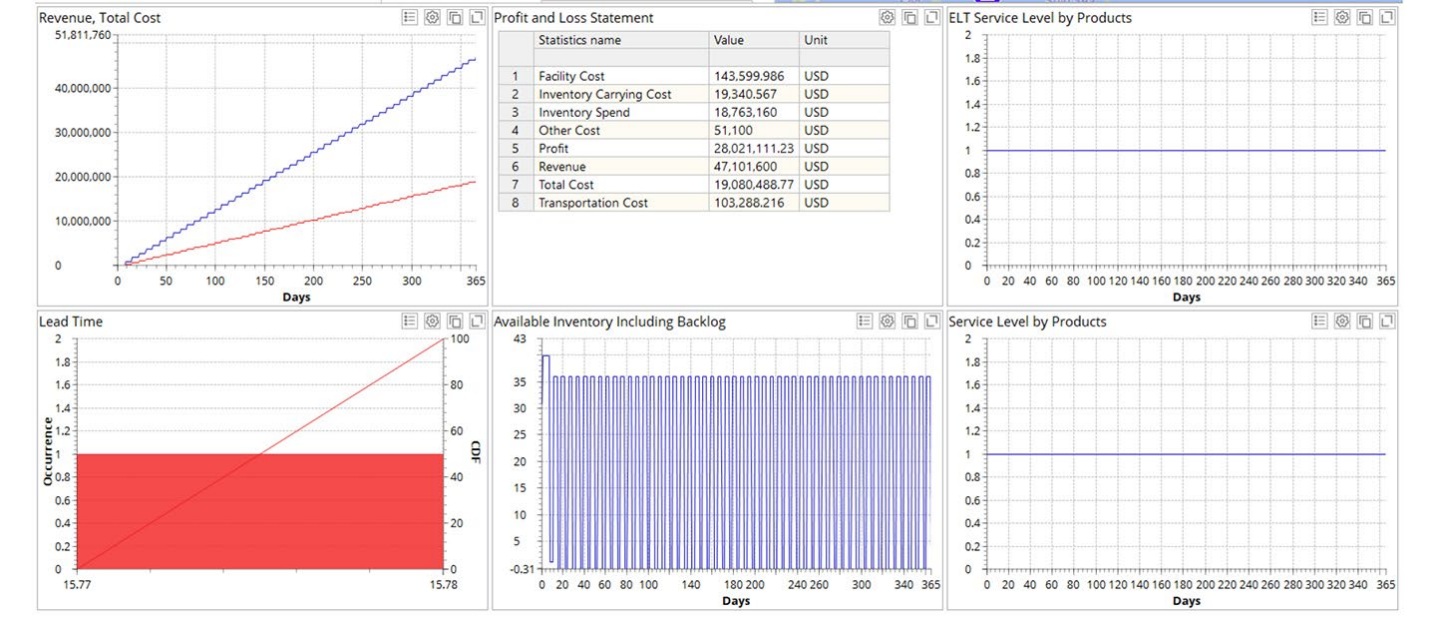
SC performance in disruption-free scenario

In Fig. 5, we illustrate the SC behaviour in a disruption-free (i.e., nominal) scenario without any disruptions.

It can be observed in Fig. 5 that the SC operates at an ELT and alpha service levels of 100% achieving a profit of $28,021 million, with a stable lead-time and balanced inventory dynamics. Now we simulate the different cases according to blackout scenarios (cf. Figure 2) and observe the gaps in SC performance as compared to the disruption-free mode (Fig. 5). In all the experiments, blackouts at the RDCs begin at March 1. The simulation period is January 1 – December 31. In case of blackout propagation, the blackout at the next stage upstream (e.g., CDC) begins the day after the blackout ends at the previous stage downstream (e.g., RDC). No blackout overlapping are considered. In case of simultaneous blackouts at different echelons (cases IIb and IIIb in Table 1), they all begin on March 1. The ELT and alpha service levels are counted as recovered service levels at the end of the simulation period.

A summary of the most interesting results of the simulation runs is presented in Table 1.

**Table 1:** Summary of computational results

	Scenario	Blackout duration at RDCs	Blackout duration at CDCs	Blackout duration at factory	Demand surge during the blackout period	ELT Service level, %	Alpha Service level, %	Alpha Service Level Change, %	ELT Service Level Change, %	Profit change,%
0	Nominal	0	0	0	0	100	100	0	0	0
1.	I	5	0	0	0	100	98	-2	0	-4.5
2.		10	0	0	0	100	98	-2	0	-4.5
3.		15	0	0	0	100	96.2	-3.8	0	-6.5
4.		5	0	0	200%	100	96.2	-3.8	0	-4.5
5.		10	0	0	200%	100	96.2	-3.8	0	-4.5
6.		15	0	0	200%	100	94.5	-5.5	0	-4.7
7.	IIa	5	5	0	0	100	98.0	-2	0	-4.5
8.		5	10	0	0	94.9	93.0	-7	-5.1	-8.4
9.		10	5	0	0	94.9	93.0	-7	-5.1	-8.4
10.		5	5	0	200%	100	96.2	-3.8	0	-4.5
11.		10	5	0	200%	98.0	94.4	-5.6	-2	-3.0
12.		10	10	0	200%	96.2	92.7	-7.3	-3.8	-1.6
13.		10	15	0	200%	94.3	90.9	-9.9	-5.7	-1.6
14.		15	5	0	200%	98.1	91.0	-9.0	-1.9	-4.8
15.		15	10	0	200%	98.0	90.9	-9.1	-2	-4.8
16.		15	15	0	200%	94.2	87.5	-12.5	-5.8	-3.6
17.	IIb	5	5	0	0	100	98.0	-2	0	-4.5
18.		10	10	0	0	100	98.0	-2	0	-4.5
19.		15	15	0	0	100	96.2	-3.8	0	-6.5
20.		5	5	0	200%	100	96.2	-2	0	-4.5
21.		10	10	0	200%	100	96.2	-2	0	-4.5
22.		15	15	0	200%	100	92.6	-7.4	0	-6.5
23.	IIIa	5	5	5	0	99.9	98	-2	-0.1	-4.9
24.		5	10	10	0	98	96.1	-3.9	-2	-5.4
25.		5	15	15	0	94.1	92.3	-7.7	-5.9	-6.1
26.		10	5	5	0	98	96.1	-3.9	-2	-5.4
27.		10	10	10	0	96	94.2	-5.8	-3	--5.8
28.		10	15	15	0	92.1	90.4	-9.6	-7.9	-6.1
29.		15	15	15	0	94	90.4	-9.6	-6	-8.3
30.		5	5	5	200%	98.0	94.4	-5.6	-2	-2.9
31.		5	10	10	200%	94.3	90.9	-9.1	-5.7	-1.0
32.		5	15	15	200%	89.0	85.9	-14.1	-11.0	+ 1.5
33.		10	5	5	200%	96.2	92.7	-7.3	-3.8	-0.1
34.		10	10	10	200%	90.7	87.5	-12.5	-0.9	+ 0.1
35.		10	15	15	200%	85.7	82.7	-17.3	-14.3	+ 3.1
36.		15	5	5	200%	98.0	90.9	-9.9	-2	-5.1
37.		15	10	10	200%	92.4	85.9	-14.1	-7.6	-1.7
38.		15	15	15	200%	87	81	-19	-13	-0,4
39.	IIIb	5	5	5	0	100	98.1	-1.9	0	-4.5
40.		10	10	10	0	100	98.1	-1.9	0	-4.5
41.		15	15	15	0	100	96.2	-3.8	0	-6.5
42.		5	5	5	200%	100	96.2	-3.8	0	-4.5
43.		10	10	10	200%	100	96.2	-3.8	0	-4.5
44.		15	15	15	200%	100	92.6	-7.4	0	-6.4

Next, we analyse the results presented in Table 1 and deduce some useful managerial implications.

#### Impact of the blackout localization vs. propagation

In this set of simulations, we run and compared scenarios for localized and propagated blackouts to understand the performance impact and if a blackout propagation creates the ripple effect in the SC. When analyzing lines 1/7/23 vs 17/39 as well as lines 3/29 vs 19/41, it can be observed that simultaneous blackouts have lower impacts on performance, resilience and viability as the sequential blackouts. Moreover, these effects amplify with an increase in disruption duration.

*Insight 1: The blackout propagation induces the ripple effect in SCs. The simultaneous blackouts create less damage for the SC performance, resilience, and viability as compared to the sequential blackouts.*

#### Impact of the blackout duration

We now compare results of different simulations concerning the impact of blackout duration. It can be observed (see e.g. lines 1, 2, and 3) that in the case of localized blackouts, the major magnitude comes even with a short blackout. The long blackouts increase the impact significantly adding the blackout duration as a contributing factor to the immediate impact.

##### Remark 1

Re-order period in the model is 1 week and so larger than the short blackout duration but shorter than medium and long lockdown durations.

The duration of the blackout plays important role in multi-echelon settings, especially in case of sequential blackouts at different echelons. Longer blackouts result in lower product unavailability (e.g., compare lines 5 vs 6 and 23 vs 29). As for the short sequential blackouts, their propagation across different SC echelons does not create additional negative performance effects (see lines 1, 7, and 23) – longer durations do increase the negative impact (lines 3 and 29). In the case of multi-echelon, sequential blackouts, the longer durations result in lower performance both for resilience and viability. This effect is slightly mitigated in case of simultaneous blackouts at different echelons.

*Insights 2. The blackout duration has implications for ecosystem viability, especially for critical and perishable products. Increase in blackout duration results in longer periods of product unavailability, especially in the case of multiple, sequential blackouts at different SC echelons.*

#### Impact of the irrational customer behavior

**Fig. 6:**
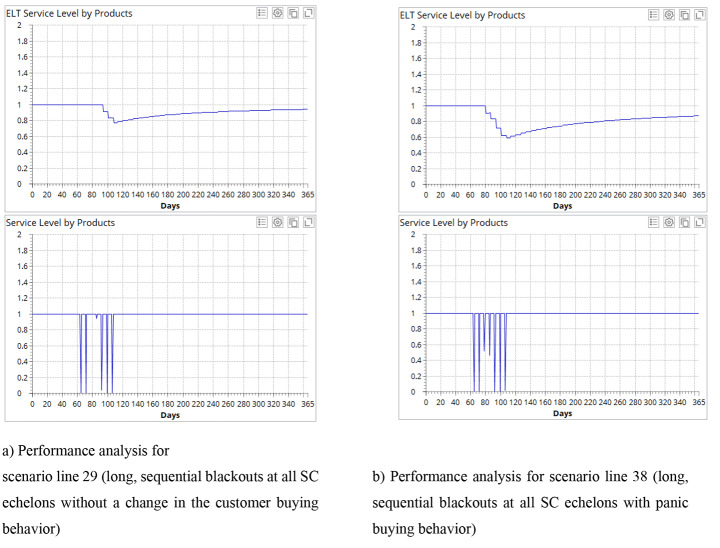
Comparison of performance with and without panic buying.

In this set of experiments, we introduce irrational customer behavior which is an immediate and strong surge in demand (200%) as a panic reaction to the blackout. It can be observed from Table 1, e.g., lines 3 vs 6 and 29 vs 38 that the panic buying influences the SC performance, resilience and viability both for localized and propagated blackouts of short, medium and long durations. The effects are mixed (Fig. 6).

On the one hand, SC economic performance benefits from the increased demand and results in higher profits. On the other hand, both ELT and alpha service levels decrease meaning the negative effect on SC resilience and viability. In case of simultaneous blackouts at different echelons, the panic buying does not have such a strong effect due to shorter total periods of blackout and high demand (see lines 38 vs 44 and 16 vs 22).

*Insight 3: Panic buying leads to decrease of product availability and on-time delivery while it can increase the profitability through high demand. The panic buying effects should be considered when developing preparedness strategies for blackout scenarios.*

## Managerial insights and future research opportunities

In this section, we generalize insights obtained from the experiments. We organize this section around three major questions:

- Is blackout a special case of SC risks? If yes, what are its specific features?
- What are the major factors/scenarios under which the blackout impacts increase, and what are possible mitigation/preparedness strategies?
- How to balance performance, resilience and viability with consideration of blackouts?

### Managerial insights

Our first observation from the simulation results is that blackout can be considered a distinct case of SC risks (Table 2).

**Table 2:** Instantaneous supply chain disruptions, blackout and pandemic

	Instantaneous disruption, e.g., an earthquake or fire	Blackout	Pandemic
Performance Impact	Instant impact	Instant and medium-term impact	Long-lasting impact with hardly predictable scaling
Scope	Single supply chain echelon (with possible propagations)	Local disruption or simultaneous disruptions in supply, demand, and logistics	Simultaneous disruptions in supply, demand, and logistics
Customer behaviour	Rational/Irrational	Irrational	Irrational
Recovery	Begins when disruption is over	Can begin only when the power is available again	Is performed in the presence of a disruption and its unpredictable scaling
Timing	A single disruptive event	A single or multiple disruptive events leading to simultaneous and sequential closures of suppliers, facilities and markets	Simultaneous and/or sequential openings and closures of suppliers, facilities and markets
Society impact	Resilience of individual supply chains	Viability of the ecosystems	Viability of the ecosystems

Blackout risk has some similarities with both instantaneous disruptions, e.g., an earthquake or fire and pandemics and combines their different individual features into a unique set of features which cannot be observed in other SC risks (Shen & Li, 2017; Sodhi et al., 2021). Blackout is characterized by both an immediate and medium-term impact. It can lead to both local disruptions or simultaneous disruptions in supply, demand, and logistics accompanied by irrational customer behavior, e.g., a panic buying. We note that we use the term “irrational” meaning a comparison to the normal, rational customer behavior which presumes some predicted product availability. In terms of buying behavior under the unpredictable product availability, such a panic buying behavior can be seen rational under crisis conditions similar to long-term product shortages caused by instantaneous disruptions. For example, rice production in Australia was reduced by 98% after a long drought period in 2008. As a result, consumers in Vietnam, India, and Hong Kong began to stockpile rice due to the fear of insufficient future supply (Bradsher, 2008).

The recovery can begin only once the power supply is restored. It can be manifested by both single and multiple disruptive events leading to simultaneous and sequential closures of suppliers, facilities and markets. The impact of blackout can adversely affect both SC economic performance, resilience, and viability.

The specific feature of the blackout is simultaneous dynamics of two systems, i.e., energy structure and material flow structure. Power loss propagation and SC ripple effect are therefore encountered simultaneously. This is a novel and underexplored setting of cross-structural ripple effects. Among SC disruptions, blackouts have a strong social and societal component. This requires analyses of not only SC resilience but also business ecosystem viability (Ivanov 2021c). In particular, the reconfigurable SC design can be favorable to sustain severe crisis such a blackout (Dolgui et al., 2020b).

Mitigation strategies are multiple and can include facility fortification by installing backup power generators, design of low-energy consumption SCs, usage of early-warning systems, and usage of a portfolio of different trucks (diesel, electro, hydrogen). Besides, diversified energy portfolio at different warehouses can help. SCs with mono-energy structures are more prone to blackout and we stress the importance of diversifying energy sources in SC designs. Another important measure is to identify supplier proneness to power outages.

Recovery strategies depend on the timing when the blackout was recognized. One can use inventory in places served with green energy sources. Since electrical trucks can be unavailable during the blackout, switching to logistics with alternative fuel technologies (diesel or hydrogen) can help deploy plans for stabilization immediately after the power outage and mid-term recovery.

### Future research opportunities

Some new and relevant research areas arise in the blackout risk context, e.g.:

- SC design with different, alternative energy sources considering disruptions in some of them;
- Simultaneous and cross-structural ripple effects;
- Viable SC, reconfigurable SC, and SC multi-structural dynamics (Dolgui et al., 2020b).
- Perishable products and blackouts.
- Role of early warning systems, digital technology and end-to-end visibility for timely detection of blackouts and deployment of stabilization and recovery plans (Yoon et al., 2020; Dubey et al., 2021a; Dolgui & Ivanov, 2021).
- Intertwined supply networks and blackout impact mitigation (Ivanov and Dolgui 2020, Feizabadi et al., 2021).

## Conclusions

Uncertainty in SCs has often been focused on disruption risks in material flows, e.g., the impact of supplier disruptions on resilience and performance. New sources of uncertainty stem from energy-related risks which become increasingly salient in light of the geopolitical tensions and resulting risks of energy supply interruptions. In addition, transformations of energy systems towards new energy sources with less predictable or weather-dependent output create new challenges for resilient SC operations - where energy is missing, the material will be missing. While the research on energy-efficient manufacturing and logistics has been flourishing in the engineering literature for the last two decades, the SC management perspective still needs to be developed. Besides, energy perspective is almost missing in SC resilience research. In this paper, we presented the results of a simulation study on the blackout impacts on SC performance, resilience and viability. The risks of blackouts increase and this calls research community to develop methods of impact assessment, mitigation and recovery strategies. Our results show that depending on the scope of the affected areas and the blackout duration, SCs can be impacted to different extent. Through simulations using anyLogistix digital SC twin, we identified potential impacts of blackouts on SCs for scenarios of different severity. The major observation from the simulation experiments is that the dynamics of the power loss propagation across different regions, the blackout duration, simultaneous unavailability of supply and logistics along with the unpredictable customer behavior might become major factors that determine the blackout impact on the SC performance and ecosystem viability.

Our study allowed for some generalized managerial insights and revealed some new research directions. First, we conceptualized blackout as a special case of SC risks which is distinctively characterized by a simultaneous shutdown of SC processes, disruption propagations (i.e., the ripple effect), and danger of viability losses for entire ecosystems. Second, we articulated some managerial insights which can be used by decision-makers to predict the operative and long-term impacts of blackouts on the SCs and society and develop mitigation and recovery strategies. Third, we proposed new research areas related to blackout impact on SCs.

As for limitations of this study, we concisely reduced the technical complexity to make the managerial insights more depictive. Another limitation is the usual restriction on the generalization and validation of the simulation results due to their contextual data and a black box modeling approach. However, the possibility to model complex systems and deduce novel and relevant managerial insights argue in favor of the simulation method applications. Finally, the problem under consideration could be modelled using other approaches, e.g., Bayesian networks (Hosseini et al., 2020; Hosseini & Ivanov, 2019, 2021; Liu et al., 2021).

In future research, our study can be extended in several ways. We examined upstream propagation of blackouts; downstream propagation is also interested. Impact of other inventory control and sourcing control policies could be analysed. Products with a ban on the backordering can be studied. Different recovery policies for the blackout periods can be introduced that change inventory and sourcing control during and after the disruption adapting it to structural dynamics as proposed in (Ivanov, 2019; Ivanov & Rozhkov, 2020). Other degrees of irrational customer behavior (e.g., demand increase by 300%, 400% etc.) can be examined. Blackout overlappings at different echelons can also offer some new interesting insights. Finally, we point to the new digital technologies that have a potential to improve the ripple effect control in cases of blackouts (Yoon et al. 2019, Dubey et al., 2021a, b>, Ivanov 2021a, Ivanov and Dolgui 2021, Kosasih & Brintrup, 2021, Shen et al., 2021, Rai et al., 2021).
